# Recent progress on heterologous protein production in methylotrophic yeast systems

**DOI:** 10.1007/s11274-024-04008-9

**Published:** 2024-05-11

**Authors:** Masashi Tsuda, Koichi Nonaka

**Affiliations:** Biologics Technology Research Laboratories I, Daiichi Sankyo Co., Ltd., 2716-1 Kurakake, Akaiwa, Chiyoda, Gunma 370-0503 Japan

**Keywords:** *Komagataella phaffii*, Methylotrophic yeast, *Ogataea minuta*, *Ogataea polymorpha*, Recombinant protein production

## Abstract

Recombinant protein production technology is widely applied to the manufacture of biologics used as drug substances and industrial proteins such as recombinant enzymes and bioactive proteins. Various heterologous protein production systems have been developed using prokaryotic and eukaryotic hosts. Especially methylotrophic yeast in eukaryotic hosts is suggested to be particularly valuable because such systems have the following advantages: protein secretion into culture broth, eukaryotic quality control systems, a post-translational modification system, rapid growth, and established recombinant DNA tools and technologies such as strong promoters, effective selection markers, and gene knock-in and -out systems. Many methylotrophic yeasts such as the genera *Candida*, *Ogataea*, and *Komagataella* have been studied since methylotrophic yeast was first isolated in 1969. The methanol-consumption-related genes in methylotrophic yeast are strongly and strictly regulated under methanol-containing conditions. The well-regulated gene expression systems under the methanol-inducible gene promoter lead to the potential application of heterologous protein production in methylotrophic yeast. In this review, we describe the recent progress of heterologous protein production technology in methylotrophic yeast and introduce *Ogataea minuta* as an alternative production host as a substitute for *K. phaffii* and *O. polymorpha*.

**Supplementary Information:**

The online version contains supplementary material available at 10.1007/s11274-024-04008-9.

## Introduction

Recombinant protein production technologies have been widely applied to the manufacture of biological drug substances and industrial enzymes. Recently, over 200 biopharmaceuticals have been registered and approved by the US Food and Drug Administration (FDA) and the European Medicines Agency; annual sales are expected to exceed US$200 billion within the next 10 years (Kesik-Brodacka 2018). Many industrially valuable enzymes, such as phytases, lipases, mannanases, and xylanases, have been produced at a commercial scale via recombinant protein production technology (Spohner et al. 2015).

In response to the strong demands to boost such production further, several heterologous protein production systems have been established in the following representative hosts: mammalian cells such as Chinese hamster ovary cells, insect cells, yeast, and *Escherichia coli*. Among these production systems, yeast might be a particularly conventional host for various heterologous protein due to their biological safety, heterologous protein productivity, and economic advantages etc. (Gellissen 2000; Kim et al. 2015).

Since Ogata et al. first isolated methylotrophic yeast in 1969, many methylotrophic yeasts such as the genera *Candida*, *Ogataea*, and *Komagataella* have been reported (Ogata et al. 1969; Raymond et al. 1998; Cereghino and Cregg 2000; Gellissen 2000; Sasano et al. 2008). These methylotrophic yeasts have the unique property of being capable of growth utilizing methanol as the sole carbon source. Therefore, initially, they were expected to be a production host of biomass and single-cell protein (SCP) on media containing inexpensive methanol derived from oil or natural gas (Koti and Keith 1996).

In *Komagataella phaffii*, a high-cell-density fermentation process through continuous optimized culture containing methanol was developed. However, owing to the drastic increase in the cost of methanol because of the oil crisis in the 1970s, SCP production by methylotrophic yeast became less economically advantageous. Meanwhile, as an alternative usage, *K. phaffii* has been investigated since the 1980s as a heterologous protein production host (Wegner 1990). Then, alcohol oxidase gene (*AOX1*) encoding an important methanol-responsive gene, and its promoter, which is commonly used for protein production, were isolated (Ellis et al. 1985). The combination of fermentation techniques developed for SCP manufacture and a tightly regulated strong promoter served as a basis for methylotrophic yeast to be used successfully as a heterologous protein production host.

Methylotrophic yeast systems are desirable from the perspective of biological safety for commercial production. For example, the use of type C phospholipid lipase (EC 3.1.4.3), which is expressed by *K. phaffii*, for the degumming of edible vegetable oil was granted generally recognized as safe (GRAS) status by the FDA (Ciofalo et al. 2006). These systems also have several other advantages as follows: the ability to secrete proteins into culture broth; a eukaryotic protein quality control system; a post-translational modification system; and rapid growth (Hartner et al. 2008; Zhang et al. 2009).

**Fig. 1:**
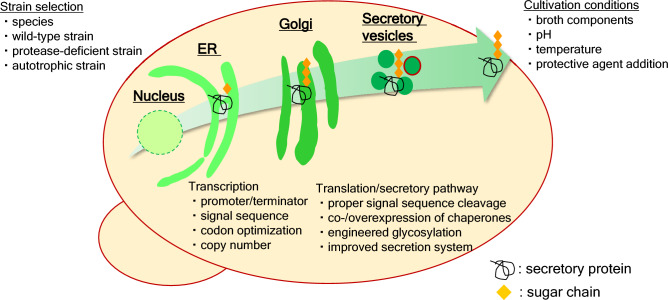
Schematic overview of protein secretory pathway: points to consider to develop a refined recombinant protein expression system in methylotrophic yeast

Against this background, there are numerous points to be considered when attempting to optimize methylotrophic yeast systems (Fig. 1). Among these points, in this review we focus on recent advances in four topics: strain selection, expression promoters, the secretory system, and protease-deficient strains. These were selected because our experience suggests that these issues are particularly important for recombinant protein productivity.

Regarding strain selection, in addition to *K. phaffii* and *Ogataea polymorpha*, which are major sources of strains among methylotrophic yeast production hosts, we would like to introduce *Ogataea minuta* as an alternative. *O. minuta* is a methylotrophic yeast that we and other researchers have investigated and developed as a recombinant production host. We believe that *O. minuta* is a noteworthy species because developed strains have been successfully and widely used to express recombinant proteins (Kuroda et al. 2007; Suzuki et al. 2017; Yoko-o et al. 2021; Tsuda et al. 2023).

## Strain selection

Many strains have been classified as methylotrophic yeast, but they vary in their characteristics. In this section of the paper, we refer to the background and properties of three methylotrophic yeast species.

## *Komagataella phaffii*

*Komagataella phaffii*, with the more common synonym *Pichia pastoris*, is a common methylotrophic yeast employed as a system for expressing recombinant proteins, biopharmaceuticals, and industrial enzymes (Cereghino and Cregg 2000). Several *K. phaffii* strains have made accessible for research and development (Table 1). All of the strains are derived from NRRL-Y 11430, which was deposited in the ARS Patent Culture Collection by Philips Petroleum (Wegner 1980).

**Table 1:** Representative methylotrophic yeasts for heterologous protein expression

Species	Description	Name	Reference
*K. phaffii*	Wild-type strains	Y-11430	NRRL^a^
		X-33	Daly and Hearn (2005)
	Strain with different ability in methanol usage	Mut^+^ (*AOX1*^+^, *AOX2*^+^)	Yin et al. (2007)
		Mut^s^ (*AOX1*^−^, *AOX2*^+^)	Yin et al. (2007)
		Mut^−^ (*AOX1*^−^, *AOX2*^+^)	Yin et al. (2007)
	Strains with deficiency in protease activity	SMD1163 strain (*his4pep4prb1*)	Fickers (2014)
		SMD1165 strain(*his4prb1*)	Fickers (2014)
		SMD1168 strain(*his4pep4*)	Fickers (2014)
	Strains with deficiency in histidine dehydrogenase	GS115(*his4*)	Vanz et al. (2012)
		KM71(∆*aox1 *∆::S*ARG4 his4 arg4*)	Charoenrat et al. (2013)
*O. polymorpha*		CBS4732(CCY38-22-2, ATCC34438, NRRL-Y-5445)	Stöckmann et al. (2009)
		DL-1(NRRL-Y-7560; ATCC26012)	Stöckmann et al. (2009)
		NCYC495(CBS1976; ATAA14754, NRLLY-1798)	Stöckmann et al. (2009)
*O. methanolica*		PMAD11(*ade2-11*)	Raymond et al. (1998)
*C. boidinii*		TK62(*ura*)	Sakai et al. (1999)
*O. minuta*	*O. minuta var. minuta* type strain	NBRC0975	NBRC^b^
		NBRC10402	NBRC^b^
		NBRC10746	NBRC^b^

^a^Northern Regional Research Laboratories

^b^National Institute of Technology and Evaluation (NITE) Biological Resource Center

*K. phaffii* was developed as a platform for SCP production by Philips Petroleum and later used as a basis for the development of a protein production system (Cregg et al. 1985). The advantages of the *K. phaffii* production system comprise high folding efficiency, high-cell-density fermentation, a strong and highly regulated expression system, genetic stability, robust protein secretion, easy downstream processing, FDA GRAS status, and suitability for the application of useful genome engineering techniques including the recently developed CRISPR/Cas9 gene editing tool (Zhu et al. 2019; Pan et al. 2022). According to Pichia.com, a web platform for posting information about the *K. phaffii* production system, over 5000 proteins have now been successfully produced by this system (Schwarzhans et al. 2017).

## *Ogataea polymorpha*

*Ogataea polymorpha* (syn. *Hansenula polymorpha*) is one of the most important industrially applied non-conventional yeasts (Gellissen 2000). It is a ubiquitous yeast species naturally isolated from spoiled orange juice, maize meal, the gut of various insect species, and soil (Kurtzman et al. 2011). *O. polymorpha* belongs to the *Saccharomycetaceae* fungal family, subfamily *Saccharomycetoideae* (Yamada et al. 1995; Kurtzman et al. 2011).

Most research has been performed with three basic strains designated as *O. polymorpha* DL-1, CBS4732, and NCYC495 (Table 1). These strains have independent origins and unclear relationships, while exhibiting different features including different numbers of chromosomes. Depending on the strain and separation conditions, between two and seven chromosomes can be distinguished (Marri et al. 1993). Strain CBS4732 (syn. ATCC34438, NRRL-Y-5445; CCY38-22–2) was originally isolated from soil irrigated with distillery effluent in Pernambuco, Brazil (Morais and Maia 1959). Meanwhile, the DL-1 strain (syn. ATCC26012, NRRL-Y-7560) was isolated and characterized from a soil sample (Levine and Cooney 1973). These two strains are those most widely utilized industrially. NCYC495 has been extremely widely used in the laboratory since it was isolated from concentrated orange juice in Florida (Kurtzman et al. 2011).

*O. polymorpha* is an attractive recombinant production host because this system has several advantages, including suitability for the application of genetic engineering tools, FDA GRAS status, high-cell-density fermentation, thermotolerance (capable of growing at temperatures ranging from 30 to 50 °C), ability to use multiple carbon sources, and relatively low hyper-mannosylation. Therefore, the *O. polymorpha* production host system is suitable for human-like glycoproteins and extensively used for the production of therapeutics and antivirals (Manfrão-Netto et al. 2019).

## *Ogataea minuta*

*Ogataea minuta* is a methylotrophic yeast that belongs to the *Ogataea* clade, closely related to *O. polymorpha* (Tsuda et al. 2023). Three strains, as described below, have been classified as *O. minuta* var. *minuta*, which have been deposited at the National Institute of Technology and Evaluation (NITE) as the Biological Resource Center, NITE (NBRC) collection (https://www.nite.go.jp/en/index.html) (Table 1).

One of these strains is NBRC 0975, derived from NRRL Y-441 in the Agricultural Research Service (ARS) Culture Collection at the Northern Regional Research Laboratory (NRRL), which was isolated from fermented mushrooms (Wickerham 1951). Another of these strains is NBRC 10402, derived from ATCC 26176 in the American Type Culture Collection (ATCC), which was isolated from a rotten azalea flower by Oki et al. (1972). The third strain, NBRC 10746, is derived from DSM 70718 in the German Collection of Microorganisms and Cell Cultures (DSMZ), which was isolated from forest soil by Henninger and Windisch (1975).

These strains were historically deposited as *O. minuta* var. *minuta* in the NBRC collection. However, in a phylogenetic tree created by neighbor-joining analysis based on sequence analysis of the long LSU D1/D2 domain of each strain, NBRC 10402 and NBRC 10746 were grouped into different clusters far from NBRC 0975, a type strain of *O. minuta* var. *minuta*, indicating that NBRC 0975 is quite different from NBRC 10402 and NBRC 10746. Among these strains, we have reported that NBRC 10746 is the most attractive option as a parental strain for further development of an optimal host strain because of the high productivity per cell and adequate cell mass (Tsuda et al. 2023). Genetically engineered *O. minuta* has been adopted as a recombinant production host for several proteins including novel reporter proteins, enzymes, and antibodies (Kuroda et al. 2006; Suzuki et al. 2017; Yoko-o et al. 2021; Tsuda et al. 2023).

## Expression promoters

Several promoters are now available for use in methylotrophic yeast production systems. In *K. phaffii*, the constitutive glyceraldehyde-3-phosphate dehydrogenase (*KpGAP*) promoter and the methanol-inducible alcohol oxidase I (*KpAOX1*) promoter are the most popular (Yan et al. 2022a, b). For optimizing recombinant protein production, the selection of an appropriate promoter is critical. For example, when the recombinant protein of interest is toxic, an inducible promoter is preferable because this enables avoidance of stress on the cells caused by accumulation of the expressed protein during the growth phase (Ahmad et al. 2014). In this section of the paper, we describe recent advances in unique promoters adopted in methylotrophic yeast production systems.

## Constitutive promoters

Generally, constitutive promoters are adopted for the stable and continuous production of recombinant proteins during fermentation. In *K. phaffii*, it has been reported that the *KpGAP*, *KpGCW*, *KpTEF*, and *KpPGK* promoters are major constitutive promoters (Yan et al. 2022a, b). Among these, the *KpGAP* promoter has been commonly used as a strong promoter. However, it was reported that the expression level associated with the *KpGAP* promoter decreased by two-thirds in glycerol-containing medium and one-third in methanol-containing medium (Cereghino and Cregg 2000). Therefore, there has been demand for alternative constitutive promoters and those promoters that have been developed have been reviewed (Yan et al. 2022a, b).

The *KpGCW* promoter was proposed as a stronger promoter than the *KpGAP* one. Indeed, it was reported that EGFP production by the *KpGCW* promoter was tenfold and fivefold higher than that by the *KpGAP* promoter, in glycerol-containing or methanol-containing medium and glucose-containing medium, respectively (Ahmad et al. 2014). Meanwhile, via a random mutagenesis-based approach, genetic engineering of the *KpGAP* promoter was attempted to generate a functional *KpGAP* promoter library featuring a wide range of activity. This resulted in several stronger promoter variants being developed, the strongest of which showed activity almost 20-fold that of the wild-type promoter (Qin et al. 2011). Furthermore, a regulatory library of the *KpGAP* promoter was obtained by engineering of the putative transcription factor binding genome region, with activity ranging from 0.4- to 3.1-fold higher than that with the original promoter (Ata et al. 2017).

In *O. polymorpha*, the *OpGAP* promoter was the first identified and the only major constitutive promoter (Heo et al. 2003). Recently, in response to demand for alternative constitutive promoters, Yan et al. (2022a, b) characterized 10 constitutive promoters. Among these, the *OpGCW* promoter showed 1.3-fold higher activity than the *OpGAP* promoter in glucose-containing medium and stable strong activity with various carbon sources (Yan et al. 2022a, b). In *O. minuta*, the *OmGAP* and *OmPGK* promoters are available (Suzuki et al. 2017; Yoko-o et al. 2021). Kuroda et al. (2006) evaluated the activity of β-galactosidase expressed by the *OmGAP* and *OmAOX* promoters in glucose-containing, glycerol-containing, and methanol-containing media. The *OmGAP* promoter constitutively expressed with any carbon sources. By contrast, the *OmAOX* promoter was inducible with only methanol (Kuroda et al. 2006).

## Inducible promoters

The availability of methanol-inducible promoters is one of the most advantageous features of methylotrophic yeast production systems. Methylotrophic yeast species share the methanol utilization (MUT) pathway, which has been well refined and integrated to assimilate and dissimilate methanol (Hartner and Glieder 2006). In *K. phaffii*, KpAOX1 is the first-step enzyme in the MUT pathway to oxidize methanol to formaldehyde (Yan et al. 2022a, b). Since first being employed for recombinant protein production in 1987, the *KpAOX1* promoter is used as an attractive promoter in *K. phaffii* due to its unique feature of being highly suppressed by glucose and glycerol and strongly induced by methanol (Tschopp et al. 1987; Ahmad et al. 2014).

The regulatory mechanism of *KpAOX1* was investigated, which resulted in the identification of cooperative active transcription factors, KpMIT1, KpMXR1, and KpPRM1 (Wang et al. 2016a, b). KpMXR1 plays a critical role as the regulator of some genes involved in methanol utilization and other regulatory systems (Lin-Cereghino et al. 2006). By the overexpression of KpMXR1 and following inhibition of glycerol transporter 1, the expression level of *KpAOX1* was shown to be upregulated in glycerol-containing and glycerol plus methanol-containing media (Zhan et al. 2017). The synthetic positive feedback circuit of Mxr1 driven by a weak *KpAOX2* promoter enhanced recombinant protein production efficiency (Chang et al. 2018). Furthermore, a synthetic *KpAOX1* promoter library was generated by deleting or duplicating transcription factor binding sites, showing a 1.6-fold higher protein production level than the native promoter (Hartner et al. 2008).

A synthetic core promoter and variants that were designed using a consensus sequence were developed to generate a library with diverse sequences exhibiting different properties (Vogl et al. 2014). Promoter engineering by high resolution systematic mutagenesis was performed to reveal the *AOX1* core promoter sequence (Portela et al. 2018). To regulate expression of the *AOX1* promoter, manipulating poly (dA:dT) tracts was conducted to generate 34 variants with n 0.25 and 3.5 fold of the wild-type promoter activity (Yang et al. 2018). Besides the *KpAOX1* promoter, to identify alternative methanol-inducible promoters, transcriptional expression analysis was conducted in *K. phaffii* under various carbon sources. Fifteen different promoters, which are involved in the MUT pathway and response to methanol at different expression levels, were identified. Among these promoters, the promoter of *KpCAT*, which is a gene encoding an enzyme that catalyzes the conversion of hydrogen peroxide produced by methanol oxidation into water and oxygen in methanol metabolism, showed strong methanol induction and the highest expression level. The *KpCAT* promoter can also be induced by oleic acid at a similar expression level to methanol. Therefore, the *KpCAT* promoter would be a promising alternative inducible promoter in methanol-deficient conditions (Vogl et al. 2018).

Vogl et al. (2020) introduced orthologous promoters derived from a related methylotrophic yeast species, *O. polymorpha*, into *K. phaffii*. The promoter of *OpMOX*, which is a homologous gene to *KpAOX1,* and the promoter of *OpFMD*, which is a gene encoding formate dehydrogenase, presented production levels similar to and threefold higher than that of *KpAOX1*, respectively. These results suggested that orthologous promoters in other eukaryotic hosts sometimes surpass endogenous promoters due to an otherwise unobtainable regulatory control (Vogl et al. 2020).

In *O. polymorpha*, several methanol-inducible promoters are available for heterologous protein production, such as *OpMOX*, *OpFLD*, and *OpDAS* (the promoter of the dihydroxyacetone synthase gene) (Manfrão-Netto et al. 2019). These promoters show similar features to the *KpAOX1* promoter in terms of strength and inductivity; on the other hand, they are quite different in terms of transcriptional regulation in response to carbon sources. As described above, the expression of *KpAOX1* was strictly suppressed by glucose and glycerol, while the expression of *OpMOX* was not suppressed by glycerol (Manfrão-Netto et al. 2019).

Recently, 22 promoters, which are involved in the MUT pathway, precursor supply pathway, and reactive oxygen defense pathway, were identified for fine-tuning protein production in *O. polymorpha* (Yan et al. 2022a, b). In *O. minuta*, the *OmAOX* promoter and other MUT-related promoters, including the *OmDAS* and *OmFDH* promoters, are available for heterologous protein production same to other methylotrophic yeast (Kuroda et al. 2006). Yoko-o et al. (2021) revealed that the *OmAOX* promoter in *O. minuta* was suppressed by glycerol as well as glucose, and was similar to *KpAOX1* in terms of responsiveness to a carbon source, although *O. minuta* was closely related to *O. polymorpha* taxonomically. We cloned three *OmAOX* promoters from three *O. minuta* strains, evaluated the heterologous protein production ability of these three strains, and reported that NBRC 10746 showed the best performance (Tsuda et al. 2023).

## Secretory system

One of the major advantages of using *K. phaffii* as a production host is its ability to secrete recombinant protein with high titer, proper folding, post-translational processing, and biological activity into the culture broth (Ahmad et al. 2014). The initial step of the secretory pathway is the transport of a newly synthesized protein into the endoplasmic reticulum (ER). Upon entering the ER, the protein undergoes several modifications including signal peptide removal, *N*-glycosylation, and disulfide bond formation to fold into its native state. When the protein is misfolded or aggregated in the ER, the unfolded protein response (UPR) pathway, which is responsible for the induction of genes involved in protein folding, is triggered. In parallel to the UPR pathway, ER-associated degradation by the proteasome resolves clogged protein secretion pathways. Therefore, proper folding of proteins in the ER is quite important for the efficient production of recombinant proteins (Damasceno et al. 2007, 2012; Braakman and Hebert 2013). In this section of the paper, we provide an overview of the topic of post-translational modification, including secretion signal sequences, folding chaperones, and engineered glycosylation.

## Secretion signal sequences

A signal peptide usually fused at the N-terminal of a newly synthesized protein plays an important role in directing the secretion of the protein. The alpha-mating factor pre-pro leader sequence (α-MF) derived from *S. cerevisiae* is the most commonly employed signal peptide in several yeast recombinant production systems, including *K. phaffii*, *O. polymorpha*, and *O. minuta* (Damasceno et al. 2012; Manfrão-Netto et al. 2019; Tsuda et al. 2023). α-MF is composed of two domains: the pre-region that is responsible for directing the nascent protein into the ER, where it is then cleaved by signal peptidase, and the pro-region, which plays a role in transfer of the protein from the ER to the Golgi apparatus where it is finally cleaved by the endoprotease KEX2. The remaining two Glu-Ala repeats in the pro-peptide are trimmed by STE13 to generate mature α-MF (Julius et al. 1983).

While α-MF was adapted as a secretion signal, it was commonly reported that the N-terminal of the recombinant protein was non-homologous due to incomplete STE13 processing. To improve the processing of α-MF, co-overexpression of HAC1, a transcription factor in the UPR pathway, with the membrane protein adenosine A2 receptor was conducted (Guerfal et al. 2010). Furthermore, α-MF has been refined through codon optimization, directed evolution, addition of spacer sequences, and site-directed mutagenesis. Among generated variants, deletion of amino acids 57–70, corresponding to the predicted 3rd alpha helix of α-mating factor secretion signal, increased secretion of reporter proteins (Lin-Cereghino et al. 2013; Duan et al. 2019). Meanwhile, there is a possibility that α-MF causes protein aggregation, limiting export from the ER. Therefore, a hybrid secretion signal possessing the *S. cerevisiae* OST1 signal sequence followed by the α-MF pro-region was proposed to facilitate co-translational translocation into the ER (Barrero et al. 2018). As an alternative secretion signal peptide, several native signal sequences derived from highly secreted proteins accomplished the secretion of a recombinant protein (Damasceno et al. 2012). Recently, several endogenous signal peptides including DAN4, GAS1, MSB2, and FRE2 were identified via secretome and genome analyses in *K. phaffii* (Duan et al. 2019).

## Folding chaperones

A possible strategy to promote the processing and folding of proteins, to increase the yield and quality of recombinant protein production, is reinforcement of the function of ER-resident chaperones. In *K. phaffii*, it has been reported that increased production or secretion of a recombinant protein was achieved by co-expression of the following ER chaperones: a homolog of the mammalian immunoglobulin-binding protein (BiP/KAR2), protein disulfide isomerase (PDI), redox control and oxidative stress enzymes including ERO1, GPX1, AHA1, PRX1, YAP1, or YPT6, heat shock proteins such as Dnaj, Peptidyl prolyl cis–trans isomerase (PPI), the UPR transcription factor HAC1, the kinase/RNase IRE1, or newly identified co-chaperones (Huangfu et al. 2016; Raschmanová et al. 2021). Recently, three endogenous folding factors, MPD1, a member of the PDI family; PDI2, a protein disulfide isomerase; and SIL1, a nucleotide exchange factor for BiP/KAR2, were identified by prediction based on an analysis of the secretome and genome of *K. phaffii*. Among these factors, co-expression of SIL1, which is required for protein translocation into the ER, showed the most significant effect of achieving a 3.3-fold increase of protein production (Duan et al. 2019). Suzuki et al. (2017) overexpressed genes cloned from *O. minuta*, including OmERO1, OmEUG1, OmHSP104, OmKAR2, OmMPD1, OmPDI1, and OmSCJ1, which encode proteins homologous to chaperones of *S. cerevisiae*, and evaluated their effects of increasing the production of active and intact antibodies. By the co-expression of three selected chaperones, namely, OmERO1, OmKAR2, and OmPDI1, which showed positive effects in a previous study, the resulting strain achieved 16-fold higher antibody production than the control parental strain (Suzuki et al. 2017).

## Engineered glycosylation

Glycosylation is an important post-translational modification involved in the proper folding and physiological activity of proteins (Fidan and Zhan 2015). Although the yeast system was expected to be an effective recombinant protein production host, the sugar chains produced in yeast are composed of mannose polymers (hyper-mannosylation), which is quite different from the case in humans and is associated with a shorter half-life in serum with strong immunogenicity in mammals (Ballou 1990; Khandekar et al. 2001).

In this section of the paper, we describe various efforts aimed at humanization of the glycosylation pathway in yeast to overcome this drawback. Regarding *N*-glycosylation, since *OCH1* encoding α-1,6-mannosyltransferase, which is the initial enzyme of the hyper-mannosylation pathway, was identified in *S. cerevisiae*, an *OCH1*-, *MNN1*-, and *MNN4*-deficient strain was developed. *MNN1* is the gene encoding the α-1,3-mannosyltransferase that elongates the outer-chain and inner-core oligosaccharides. *MNN4* is the gene encoding a deduced mannosyltransferase and a positive regulator of another mannosylphosphate transferase. The resulting triple-gene-knockout strain can express an intermediate *N*-glycan structure, Man5GlcNAc2, identical to the human-type structure (Nagasu et al. 1992; Chiba et al. 1998). A similar *OCH1* deletion approach was attempted in *K. phaffii*, *O. polymorpha*, and *O. minuta* (Kuroda et al. 2006; Hamilton and Gerngross 2007; Cheon et al. 2012). For further humanization of the *N*-glycosylation pathways in *K. phaffii*, screening of combinatorial libraries, consisting of anchored domains of Golgi- and ER-localized proteins and catalytic domains of several glycosyltransferases and glycosidases from many species, was conducted. The resulting strain, which lacked *KpOCH1* and has five properly localized active eukaryotic proteins, namely, mannosidases I and II, *N*-acetylglucosaminyl transferases I and II, and uridine 5ʹ-diphosphate (UDP)-*N*-acetylglucosamine transporter, successfully expressed human-type *N*-glycans *N*-acetylglucosamine2-mannose3-*N*-acetylglucosamine2 (GlcNAc2Man3GlcNAc2) (Hamilton et al. 2003).

As another approach, “GlycoSwitch” technology, which consists of *KpOCH1* disruption and the stepwise introduction of heterologous glycosylation enzymes, was proposed to convert the wild-type strain into a genetically engineered strain that modified its glycoproteins with asialo complex-type *N*-glycans Gal2GlcNAc2Man3GlcNAc2 (Jacobs et al. 2009). In *O. polymorpha*, recombinant glycoprotein production was attempted, with the resulting glycoprotein showing 27% lower glycosylation than the glycoprotein expressed in *S. cerevisiae*. In addition, this glycoprotein with low glycosylation expressed in *O. polymorpha* was not recognized by anti-α-1,3-mannose antibody, although the control glycoprotein expressed in *S. cerevisiae* was positively detected (Kim et al. 2004). These results suggested the possibility that the recombinant glycoprotein expressed in *O. polymorpha* was not immunogenic (Ballou 1990). Less mannosylation in *O. polymorpha* would be due to the fact that the glycosylation pathway did not add α-1,3-linked residues (Kunze et al. 2009).

In the last few decades, recombinant glycoprotein production systems using genetically engineered *O. polymorpha* strains have been developed, with the resulting strains lacking important genes that encode enzymes involved in the hyper-mannosylation pathway: *OpOCH1* and *OpALG1* encoding dolichyl-phosphate-mannose-dependent α-1,3-mannosyltransferase. However, these strains harbor the human gene encoding β-1,2-*N*-acetylglucosaminyltransferase I. The hybrid-type glycans with a monoantennary *N*-acetylglucosamine (GlcNAc1Man5GlcNAc2 and GlcNAc1Man3GlcNAc2) were successfully expressed in glycol-engineered *O. polymorpha* strains (Kim et al. 2006). Kuroda et al. (2006) chose *O. minuta* as a production system to minimize the steps in the disruption and introduction of genes related to sugar chain processing because, as revealed by nuclear magnetic resonance analysis, the assumed sugar chain structure of *O. minuta* was simpler than that of *S. cerevisiae*. The resulting genetically engineered *O. minuta* strain lacked *OmOCH1* and contained α-1,2-mannosidase, producing a human-compatible glycoprotein Man5GlcNAc2. Whether there were any *OmMNN1* gene homologs in *O. minuta* was unclear. This indicated that it was possible to retain the step of gene disruption of *OmMNN1*, encoding α-1,3-mannosyltransferase, as an advantageous feature of *O. minuta* (Kuroda et al. 2006).

In contrast to *N*-glycosylation, engineering of *O*-glycans is more challenging because the *PMT* gene, encoding *O*-mannosyltransferases as the initial reaction enzyme of *O*-mannosylation, is essential for yeast cell survival. Recently, a sialylated α-dystroglycan-type *O*-linked glycan was produced by co-expression of an α-1,2-mannosidase and protein-*O*-linked-mannose β-1,2-*N*-acetylglucosaminyltransferase I in *K. phaffii*, which previously underwent genetic engineering of the *N*-linked glycosylation pathway (Hamilton et al. 2013). When antibody production was attempted in *O. minuta*, abnormal *O*-mannosylation on the secreted antibody was detected. By adding an inhibitor of PMT1 activity [5-(3,4-bis-phenylmethoxyphenylmethylene)-4-oxo-2-thioxo-3-thiazo-lidineacetic acid; R3A-1c], this modification was partially suppressed (Kuroda et al. 2008).

## Protease-deficient strains

Yeast heterologous protein production systems are well known for high production yields, but it was also reported that yeast systems sometimes present strong protease activity, which may cause degradation of the expressed protein. The yeast vacuole in particular contains several proteases, such as proteinase A, proteinase B, carboxypeptidases, and aminopeptidases, which are responsible for extensive protein degradation (Hazel et al. 1996). To suppress protease activity, several approaches have been attempted, including changing the cultivation conditions, pH, temperature, and so on, or adding casamino acids and peptone (Werten et al. 1999). However, in some cases, it remained difficult to completely prevent proteolytic degradation. Therefore, the protease-deficient host strain was proposed to achieve high productivity of intact recombinant protein and employed for commercial production (Gleeson et al. 1998; Fickers 2014; Wang et al. 2016a, b). In this section of the paper, we describe protease-deficient strains.

In many cases, several proteases are responsible for the degradation of expressed proteins. Therefore, multiple-protease-deficient strains are recommended to prevent degradation (Table 1). Among them, the *PEP4-* and *PRB1*-knockout strains are still the most effective and widely employed (Ahmad et al. 2014). In *O. polymorpha*, the *PEP4* gene encoding proteinase A was cloned and characterized (Bae et al. 2005). In *O. minuta*, it was not clear what kinds of protease-deficient hosts were consistently effective in producing heterologous proteins. Kuroda et al. (2007) revealed that *PEP4*, *PRB1*, and *YPS1* protease-deficient *O. minuta* strains were effective for intact antibody production. However, protease-deficient strains generally show slower growth rates, lower transformation efficiency, greater susceptibility to mechanical stresses, and reduced viability (Lin-Cereghino and Lin-Cereghino 2007). Hence, we were interested in the essential protease that is most responsible for protein degradation in the *O. minuta* production system.

**Fig. 2:**
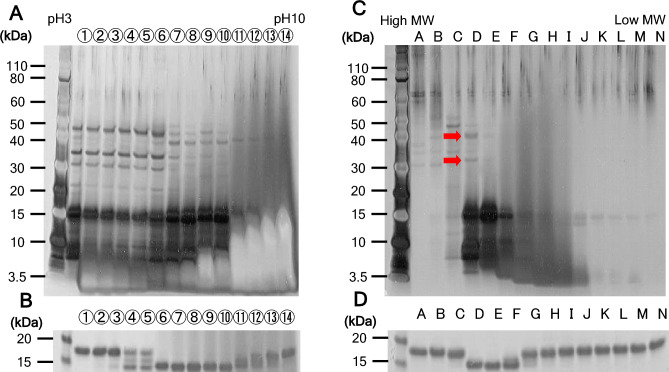
**A** Silver staining/SDS-PAGE analysis under reducing conditions of proteins fractionated by isoelectric focusing. **B** CBB staining/SDS-PAGE analysis under reducing conditions of protein degradation assay samples. **C** Silver staining/SDS-PAGE analysis under reducing conditions of fractions by size exclusion chromatography from protein pool of proteolytic activity showing lanes (Fig. 2A, lanes 6–10). Red arrows indicate the MS-analyzed protein band. **D** CBB staining/SDS-PAGE under reducing conditions of protein degradation assay sample

**Fig. 3:**
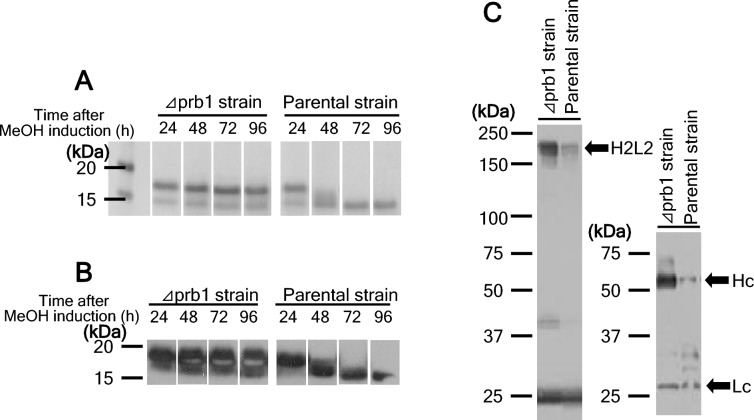
**A** CBB staining/SDS-PAGE analysis under reducing conditions of secreted recombinant protein. **B** Western blot/SDS-PAGE analyses under reducing conditions of secreted recombinant protein. **C** Western blot/SDS-PAGE analyses under non-reducing (Left) and reducing (Right) conditions of secreted antibody

Since the limited proteolysis was investigated against the model recombinant protein secreted in cultured broth, we tried to identify the protease from candidate proteins, which were partially fractionated using isoelectric focusing and size exclusion chromatography, by LC/MS–MS analysis. A fermented broth that contains secreted proteins including potential protease was loaded to Rotofor column (Bio-Rad Laboratories, Inc., Hercules, CA, USA) fractionated by a free-solution pH gradient. Silver staining of protein pools fractionated by isoelectric focusing showed various protein bands (Fig. 2A). The protein degradation assay against the model substrate protein was demonstrated and the limited proteolysis was detected by SDS-PAGE with CBB staining. Each fraction corresponding to lanes 6–10 showed the complete model protein degradation (Fig. 2B). Although lanes 6–10 was further purified by Superdex 75 gel filtration (Cytiva, Uppsala, Sweden), multiple protein bands were still investigated on SDS-PAGE (Fig. 2C). Lanes D-E completely showed model protein degradation in protein degradation assay (Fig. 2D). Unique bands showing molecular weights of 30 and 40 kDa that were highlighted by red arrows in Fig. 2C were analyzed by LC/MS–MS and identified as Prb1 protein. As a result, *OmPRB1* protein was certainly identified from the partially purified fraction. *OmPRB1*-coding region was cloned and the *OmPRB1*-deficient strain was successively generated for further studies. A degradation of secreted proteins including model protein expressed in vivo that was cleaved in the parental strain tended to be suppressed in *OmPRB1*-deficient strain (Fig. 3A, B). Additionally, degradation of a secreted IgG1-model antibody—especially heavy chain degradation in the parental strain almost prevented in the *OmPRB1*-deficient strain (Fig. 3C). The *OmPrb1*-deficient strain could grow as well as the parental strain (data not shown). These results suggested that the *OmPrb1*-deficient strain was a suitable production host in *O. minuta*. In contrast, in *K. phaffii*, 28 multi-protease-deficient strains were created and showed robust growth behavior (Ahmad et al. 2014).

## Conclusion

Methylotrophic yeasts are employed as reliable hosts for producing many recombinant proteins, from the preparation of laboratory test samples to the manufacturing of industrial products including biopharmaceuticals and enzymes. However, to express recombinant proteins of suitably high quality and yield, some issues remain to be resolved. In particular, practical trying out of strain selection using actual protein of interest might be the better way to achieve high productivity considering that the productivity in one strain can be quite good, that in another may not be. For example, the comparison of full-length antibody production levels demonstrated by several methylotrophic yeasts was shown as a case study (Table 2). Therefore, we proposed *O. minuta* as an alternative production host in this overview describing characteristic features of three methylotrophic species. We additionally pointed out that the methylotrophic yeast *Candida boidinii* is also well investigated and available as a recombinant production host (Yurimoto 2009) (Table 1). Owing to the differences in features among methylotrophic yeasts, the production host should be carefully selected. To resolve other issues, it would be effective to focus on the expression promoters, secretory system, protease-deficient strains, and the availability of genetically engineered strains. In addition to these approaches, optimization of culture medium, fermentation process and strain design including metabolic pathway engineering could be effective in refining the methylotrophic yeast heterologous protein production system. From a bioeconomy perspective, these yeast systems should also develop to produce the desired bioproduct with sustainable manufacturing process in an environmentally friendly manner (Tülek et al. 2021; Ergün et al. 2021, 2022; Çaloğlu and Binay 2023). Regarding gene editing techniques, the recently established CRISPR/Cas9 system has been rapidly developed and widely adopted as a powerful and accurate tool in *K. phaffii* and *O. polymorpha* (Weninger et al. 2016; Manfrão-Netto et al. 2019). Unfortunately, in *O. minuta*, the CRISPR/Cas9 system has not been established, although this is eagerly anticipated. In this review, we introduced important considerations for the optimization of recombinant production systems. We hope that this review contributes to achieving the full potential of methylotrophic yeasts for optimized heterologous protein production.

**Table 2:** Comparison of full-length antibody production levels in methylotrophic yeasts

Species	Antibody	Genotype	Titer (mg/ml)	Description	Reference
*K. phaffii*	Anti-RSV mAb	*och1*Δ, algΔ*::MNSI/GnTI/GnTII/GalT*	1600	Glycoengineed human-type *N*-glycan	Ye et al. (2011)
*O. polymorpha*	Herceptin	*och1*Δ*::pTDH3* > *HC/LC*, *yps1*Δ, *pep4*Δ, *prb1*Δ	Detectable by Western blot	Protease deficient strain	Jiang et al. (2019)
*O. minuta*	IgG1-model antibody	*och1*Δ, *pep4*Δ, *prb1*Δ, *yps1*Δ, *ura3*Δ*:: pTDH1* > *Hc*, *ade1*Δ*::pTDH1* > *Lc*	10	Protease deficient strain	Kuroda et al. (2007)
		*och1*Δ, *yps1*Δ, *ade1*Δ*::pTDH1* > *Lc*, hyg*:: pTDH1* > *Hc*	20	Protease deficient strain	Kuroda et al. (2008)
		*och1*Δ, *yps1*Δ, *ade1*Δ*::pTDH1* > *Lc*, hyg*:: pTDH1* > *Hc*	60	Protease deficient strain, R3AD^a^ was added	Kuroda et al. (2008)
		*och1*Δ, *yps1*Δ, *ade1*Δ*::pTDH1* > *Lc*, *ura*Δ*::OmPDI/OmERO1/OmKAR2*, zeo*:: pTDH1* > *Hc*	30	Protease deficient strain, R3AD^a^ was added, co-overexpression of chaperones	Suzuki et al. (2017)

^a^A rhodanine-3-acetic acid derivative, the Pmt inhibitor

### Supplementary Information

Below is the link to the electronic supplementary material.

Supplementary file1 (DOCX 39 KB)

## Data Availability

No datasets were generated or analysed during the current study.
