# Supplementary material for: Recent progress on heterologous protein production in methylotrophic yeast systems
Source: World J Microbiol Biotechnol. 2024 May 11;40(7):200. doi: 10.1007/s11274-024-04008-9 (PMC11087369; doi:10.1007/s11274-024-04008-9)
Supplement: Supplementary file 1 — Supplementary file1 (DOCX 39 KB) [file 11274_2024_4008_MOESM1_ESM.docx]

**Materials and Methods**

**Yeast strains and media**

The *O. minuta* YK1-3 strain was used as a host strain for the IgG1-model antibody production (Kuroda et al. 2006). The *O. minuta* tat06213 strain was used as a host strain for the degradation-model protein production (Tsuda et al. 2023). Synthetic dextrose agar (0.67% yeast nitrogen base without amino acids, 0.5% casamino acids, 2% glucose, 0.002% L-tryptophan) was used for regeneration of the spheroplasts after transformation. Yeast extract peptone dextrose (YPD) agar (1% Difco™ yeast extract, 2% Bacto™ peptone, 2% glucose, 0.004% adenine) was used for the general cultivation, and 2xYP-P6-GG medium (2% Difco™ yeast extract, 4% Bacto™ peptone, 0.5% glucose, 2% glycerol, 0.1 M potassium phosphate buffer, pH 6.0) was used for the production of the IgG1-model antibody and the degradation-model protein. Representative clones were stored in 17% glycerol at −80 °C.

**Chemicals and general recombinant DNA techniques**

*E. coli* JM109, restriction enzymes, and DNA modifying enzymes were purchased from Takara Bio Inc. (Kusatsu, Japan). The media, growth conditions, and general recombinant DNA techniques used in this study were in accordance with the work of Sambrook (Sambrook et al. 2001). Zeocin™ was purchased from Thermo Fisher Scientific (Waltham, MA, USA). Hygromycin was purchased from FUJIFILM Wako Pure Chemicals, Co., Ltd. (Osaka, Japan). All oligonucleotides in this study were synthesized by Fasmac Co., Ltd. (Kanagawa, Japan). Polymerase chain reaction (PCR) amplification was performed using the GeneAmp® PCR System 9700 (Thermo Fisher Scientific). Automated DNA sequencing was performed on an ABI PRISM® 3700 DNA Analyzer (Thermo Fisher Scientific). Transformation of *O*. *minuta* was performed as previously described (Suzuki et al. 2017).

**The protein degradation assay**

The test sample solution was mixed with the same volume degradation-model protein solution, which was manufactured using *E*. *coli* and incubated at 30°C for 15 hours. The resulting incubated solution was subject to gel electrophoresis and CBB staining.

**Isoelectric focusing (IEF)**

Prior to IEF, fermented culture broth of *O. minuta* strain YK1-3 was harvested and dialyzed with 50mM Tris buffer pH7.5. Desalted sample was mixed with 40% BioLyte® 3/10 Ampholyte #1631112 (Bio-Rad, Hercules, CA, USA) to 2% ampholyte concentration and loaded into the Rotofor cell (Bio-Rad). The loaded sample was fractionated at 10 W constant power for 3 hours using Rotofor according to the standard protocol provided by the supplier. After the run finished, fractions were harvested and assayed by the protein degradation assay. Also, the harvested pool was subjected to gel electrophoresis and silver staining.

**Size exclusion chromatography**

After IEF, pooled fractions which showed proteolytic activity was equivalated, concentrated, and applied to a Superdex 75 10/300 GL #17517401 (Cytiva, Uppsala, Sweden) using AKTA Explore system (Cytiva). 1-milliliter fractions were collected and assayed by assayed by the protein degradation assay. Also, the harvested pool was subjected to gel electrophoresis and silver staining.

**LC-MS/MS analysis**

The separated protein bands which showed proteolytic activity were extracted by in-gel digestion method. The solution containing separated protein bands was electrospray into an LTQ Orbitrap Velos (Thermo Fisher Scientific, Bremen, Germany) and analyzed as follows; LC-MS/MS run was performed in positive mode at a resolution of 60000 for the top ten precursor ions with a scan range of 350–1500 m/z and subjected to higher-energy collision dissociation with 29% normalized collision energy.

**Western blot analysis**

Western blot analysis for antibody was carried out according to the method of Suzuki et al (Suzuki et al. 2017).

**Protein production**

The IgG1-model antibody and production were carried out in accordance with the method of Suzuki *et al*. (Suzuki et al. 2017). Produced antibody was analyzed by Western blot and evaluated by enzyme-linked immunosorbent assay (ELISA) assay. ELISA assay was carried out in accordance with the method of Suzuki *et al*. (Suzuki et al. 2017).

The degradation-model protein production was carried out in accordance with the method of Tsuda *et al*. (Tsuda et al. 2023). Produced degradation-model protein was analyzed by gel electrophoresis and Western blot.

**References**

Kuroda K, Kobayashi K, Tsumura H, Komeda T, Chiba Y, Jigami Y (2006) Production of Man5GlcNAc2-type sugar chain by the methylotrophic yeast *Ogataea minuta*. FEMS Yeast Res 6(7):1052–1062. https://doi.org/10.1111/j.1567-1364.2006.00116.x

Sambrook JF, Russell DW (2001) Molecular cloning: a laboratory manual, 3rd ed. Cold Spring Harbor Laboratory Press, Cold Spring Harbor, New York.

Suzuki T, Baba S, Ono M, Nonaka K, Ichikawa K, Yabuta M, Ito R, Chiba Y (2017) Efficient antibody production in the methylotrophic yeast *Ogataea minuta* by overexpression of chaperones. J Biosci Bioeng 124(2):156–163. <https://doi.org/10.1016/j.jbiosc.2017.03.001>

Tsuda M, Nakatani Y, Baba S, Tanaka I, Ichikawa K, Nonaka K, Ito R, Yoko-o T, Chiba Y (2023) Selection of the methylotrophic yeast *Ogataea minuta* as a high-producing host for heterologous protein expression. J Biosci Bioeng 135(3):196–202. <https://doi.org/10.1016/j.jbiosc.2022.12.006>
